# Defining Disease Modification in Chronic Obstructive Pulmonary Disease

**DOI:** 10.1080/15412550902918402

**Published:** 2009-05-27

**Authors:** David M.G. Halpin, Donald P. Tashkin

**Affiliations:** 1 Royal Devon and Exeter Hospital, Barrack Road, Exeter, EX2 5DW, UK (david.halpin@rdeft.nhs.uk); 2 David Geffen School of Medicine at UCLA, Los Angeles, California 90095-1690, USA (dtashkin@mednet.ucla.edu)

**Keywords:** Chronic obstructive pulmonary disease, Disease markers, Disease modification

## Abstract

Chronic obstructive pulmonary disease (COPD) is a debilitating condition characterized by airflow limitation that is not fully reversible. It is a major cause of morbidity and mortality and represents substantial economic and social burden throughout the world. A range of interventions has been developed that decrease symptoms and address complications associated with COPD. However, to date few interventions have been unequivocally demonstrated to modify disease progression. Assessment of the potential for interventions to modify disease progression is complicated by the lack of a clear definition of disease modification and disagreement over appropriate markers by which modification should be evaluated. To clarify these issues, a working group of physicians and scientists from the USA, Canada and Europe was convened. The proposed working definition of disease modification resulting from the group discussions was “an improvement in, or stabilization of, structural or functional parameters as a result of reduction in the rate of progression of these parameters which occurs whilst an intervention is applied and may persist even if the intervention is withdrawn”. According to this definition, pharmacologic interventions may be considered disease-modifying if they provide consistent and sustained improvements in structural and functional parameters. Smoking cessation and lung volume reduction surgery would both qualify as disease-modifying interventions.

## INTRODUCTION

Chronic obstructive pulmonary disease (COPD) is a preventable and treatable disease characterized by airflow limitation that is not fully reversible. It is currently the fourth-leading cause of death in the world, and both the worldwide prevalence and mortality associated with COPD are anticipated to increase in the coming decades (1).

While current pharmacotherapy decreases symptoms and addresses complications, no interventions have been unequivocally demonstrated to modify disease progression. However, the ability to determine whether a drug modifies disease progression is complicated by the absence of a clear definition of disease modification and disagreement over appropriate markers by which modification should be evaluated. To address these issues, a working group of respiratory physicians from the USA and Europe was convened to discuss the evolving concept of disease modification in COPD. The list of participants is presented in the acknowledgements. This review publication reflects the opinions of the two authors, but may not necessarily reflect those of the wider group.

## WHAT IS DISEASE PROGRESSION AND MODIFICATION IN COPD?

**Figure 1:**
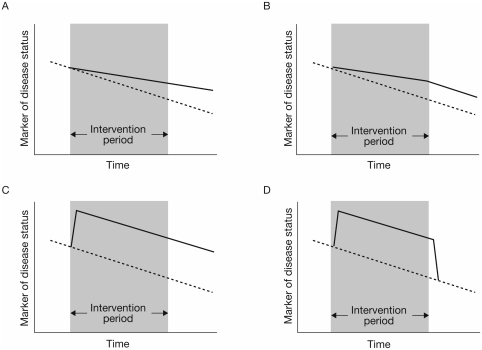
Schematic illustration of effects of an intervention on disease progression. Broken lines detail natural progression of a marker of disease progression; solid lines detail the change in this marker resulting from the intervention. (A) an intervention leading to a sustained change in the rate of progression that is maintained even after the intervention ceases; (B) an intervention leading to a sustained change in the rate of progression that occurs only during the time the therapy is administered; (C) an intervention that leads to a sustained improvement in a marker of disease status but has no effect on the rate of change of that marker over time; (D) an intervention that improves the marker of disease status during the time it is administered but provides no lasting effect on that marker once it is stopped.

No agreed definition of disease modification in COPD currently exists. One generic dictionary definition includes “… any of the changes in a disease state that are caused by an intervention”. This definition falls short of distinguishing temporary drug effects from those that affect the course of the disease over time. Broadly speaking a therapeutic intervention can influence the course of disease in one of four ways (Figure 1). An intervention may lead to a sustained change in the *rate* of disease progression that is maintained even after the intervention ceases (Figure 1A) or for as long as the therapy is administered (Figure 1B). Alternatively, an intervention may not affect the *rate* of disease progression but may lead to sustained functional/symptomatic improvement (Figure 1C), which effectively “turns the clock back” on the disease such that even after the intervention ceases, function/symptoms remain improved compared with the time that the intervention was introduced.

Finally, an intervention may simply improve disease status during the time it is administered (Figure 1D), thus providing no durable benefit after it is stopped. Consensus in the working group was that disease modification through pharmacotherapy should be defined only as interventions that alter the rate of disease progression (Figures 1A and 1B). The concept that interventions that “turned back the clock” should be considered disease-modifying was viewed as more controversial (Figure 1C). Interventions that only improve the marker of disease status during the time they are administered (Figure 1D) were not considered to be disease-modifying.

Having established what constitutes disease modification through pharmacologic intervention, it was necessary for the group to identify the most appropriate markers of disease status that can be used to monitor this process. A wide range of outcome measures and markers are available to ascertain the symptomatic efficacy of pharmacological interventions. The uses and limitations of these have recently been comprehensively evaluated by the American Thoracic Society/European Respiratory Society Task force on “Outcomes for COPD pharmacological trials: from lung function to biomarkers” (2). It was not the intention of our group to consider the merits and limitations of measures currently used to establish the symptomatic efficacy of pharmacological interventions, but rather to determine which of these measures would be appropriate to establish whether an intervention was modifying the disease process.

## BIOMARKERS FOR DISEASE PROGRESSION IN COPD

The physiologic hallmark of COPD is expiratory flow limitation and traditionally, disease progression has been measured by the decline over time in forced expiratory volume in 1 second (FEV_1_). However, in light of a better understanding of the multidimensional nature of COPD, progression might also be measured by the rate of change of other outcomes. Surrogate markers of progression could include those of (a) pathobiology, (b) physiologic indices, (c) patient-centered outcomes, and, ultimately, (d) mortality (Table 1). It is important to note that interventions that modify disease would be likely to modify the rate of change of one or more of the markers.

**Table 1:** Potential outcome measures that could be key markers of disease modification in COPD

Outcome measure	Method	Potential to measure disease progression	Practicality
**Pathophysiologic**			
Macrophages, neutrophils, CD4^+^ and CD8^+^ lymphocytes	Bronchoalveolar lavage, endobronchial biopsy, transbronchial biopsy	Not yet clear whether this is an accurate surrogate of disease progression	Invasive – not practical beyond small-scale investigation
Airway structural components	Transbronchial biopsy	Not yet clear whether this is an accurate surrogate of disease progression	Invasive – not practical beyond small-scale investigation
Cytokines, chemokines	Induced sputum	Little is known concerning long-term reproducibility and correlation with disease progression	Relatively easy and well tolerated but induction procedure can induce neutrophilic inflammation, and sputum solubilization may interfere with radioimmune assays. Samples predominantly the larger airways and may not reflect inflammation of the small airways
NO, CO, Volatile hydrocarbons (alkanes, pentanes, ethane)	Exhaled air	NO and CO only slightly elevated in COPD and little known about correlation with disease progression	Readily accessible and repeatable but CO assay is confounded by active and passive smoking
		Ethane correlates with disease progression	Ethane assay is too complex for routine use
Oxidative products, leukotrienes, cytokines, and pH (which reflects tissue acidification due to inflammation)	Exhaled breath condensate (EBC)	Accuracy of EBC as a valid reflection of alveolar lining fluid has been questioned	Noninvasive and simple but wide variability observed due to dilution from water vapor during condensation and low concentrations of the biomarkers
IL-6, IL-8, TNF-α, and CRP	Plasma/serum sampling	Serum CRP and serum TNF-α do not correlate with disease severity. Data on serum IL-6, IL-8, and fibrinogen are insufficient and inconclusive	Readily accessible and repeatable
**Physiologic**			
FEV_1_	Spirometry	Worsens over time. Exhibits sustained improvement with pharmacologic interventions *Rate of change* not easily altered by pharmacologic interventions, which may suggest this measure is resistant to change or that pharmacologic approaches applied to date are ineffective. Historically considered by many the paradigm of disease progression	Readily accessible and repeatable
**Patient-centered outcomes**			
Dyspnea	Baseline Dyspnea Index, Transition Dyspnea Index	Worsens over time and exhibits sustained improvement with pharmacologic and nonpharmacologic interventions – could be considered a surrogate marker for disease progression	Simple to perform and repeatable, but standardization of administration in clinical trials and validity as a measure of dyspnea has been questioned. The Transition Dyspnea Index measures changes in dyspnea from the initial or baseline state
Exercise capacity	6MWD progressive cycloergometry, constant work rate submaximal exercise test	Worsens over time and exhibits variable improvement with pharmacologic and nonpharmacologic interventions – could be considered a surrogate marker for disease progression	6MWD and progressive cycloergometry show poor repeatability, learning effects and effort dependence.
			Constant work rate submaximal exercise test is reliable and repeatable
Health-related quality of life	Disease-specific questionnaires, e.g., St George's Respiratory Questionnaire	Worsens over time and exhibits variable improvement with pharmacologic and nonpharmacologic interventions. Health status measures reflect the effects of the disease rather than the disease itself – could be considered a surrogate marker for disease progression	Simple to monitor
Exacerbations	Patient/physician reports	May increase in frequency over time and exhibit reduced frequency with pharmacologic interventions; however, low frequency in mild COPD would limit utility of this measure at the early stages of disease	Relatively easy to measure, although definitions differ between clinical trials
**Mortality**			
Mortality rate	Physician report	Ultimate measure of disease progression and useful to study disease modification in populations, but not suitable for monitoring progression in an individual patient	Complicated by co-morbid conditions that could contribute to mortality independently or additionally, but not exclusively to COPD

CO, carbon monoxide; COPD, chronic obstructive pulmonary disease; CRP, C-reactive protein; FEV_1_, forced expiratory volume in 1 second; IL, interleukin; 6MWD, 6-minute walking distance; NO, nitric oxide; TNF, tumor necrosis factor.

### Pathobiology as a surrogate marker for disease progression in COPD

**Figure 2:**
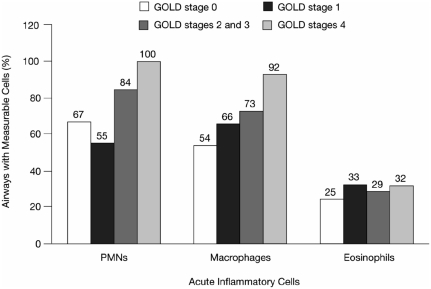
Airway inflammatory response, as measured by the percentage of the airways containing polymorphonuclear neutrophils (PMNs), macrophages, and eosinophils, among patients at each Global Initiative for Chronic Obstructive Lung Disease (GOLD) stage (4). Hogg JC, Chu F, Utokaparch S, Woods R, Elliott WM, Buzatu L, Cherniack RM, Rogers RM, Sciurba FC, Coxson HO, Paré PD. N Engl J Med 2004;350:2645–2653. Copyright © [2004] *Massachusetts Medical Society. All rights reserved*.

Since the pioneering work of Hogg and associates (3), the small airways have been recognized as the major site of airflow limitation in COPD, accounting for up to 90% of the total resistance to flow in the lungs of patients with well-established disease. Small airway walls contain neutrophils, macrophages, and both CD4^+^ and CD8^+^ T lymphocytes (but not eosinophils) and their levels progressively increase with disease severity (Figure 2) (4).

In concert with these inflammatory changes, the thickness of the airway wall structural components (epithelium, lamina propria, smooth muscle, and adventitia) also increases with disease severity. These findings suggest that it may be possible to track COPD disease progression through serial invasive evaluation of airway histopathology through endobronchial biopsies (assuming that histopathologic changes in the large airways reflect similar changes in the small airways) and transbronchial biopsies that sample the small airways and distal lung. Disease progression has been tracked in this way in asthma, but to date, this has not been performed in COPD (5). While evaluation of airway changes may be an accurate surrogate of disease progression in COPD, the invasive techniques required are not practical or feasible beyond small-scale experimental investigation. Similar concerns limit the usefulness of bronchoalveolar lavage. Thus, reliance must be placed on noninvasive pulmonary biomarkers.

Induced sputum is a relatively easy and well-tolerated procedure that can reflect inflammatory processes in the airway. Evidence for the utility of this approach in assessing inflammation has been demonstrated by comparing samples taken from smokers with those from nonsmokers (4, 6). However, induced sputum also has limitations. For example, (a) it predominantly samples the larger airways and may not reflect inflammation of the small airways; (b) the sputum induction procedure itself can induce neutrophilic inflammation; (c) solubilization of the sputum may interfere with radioimmune assays for the detection of cytokines and chemokines; and (d) little is known concerning its long-term reproducibility or its correlation with COPD severity and progression.

Exhaled air provides another readily accessible and repeatable noninvasive means for monitoring inflammation in COPD. The major exhaled gases that reflect lung inflammation include nitric oxide (NO), carbon monoxide (CO), and volatile hydrocarbons (alkanes, pentanes, ethane), the latter being markers of lipid peroxidation due to oxidative stress (7). Exhaled NO levels are commonly elevated in uncontrolled asthma, but are often normal or only slightly increased in COPD, presumably due to the conversion of NO into peroxynitrite and nitrate due to oxidative stress. On the other hand, the alveolar fraction of exhaled NO (obtained by measuring exhaled NO at different expiratory flow rates) appears elevated in COPD (8). The relationship between exhaled NO and COPD severity, progression, and acute exacerbations, however, remains to be determined. Exhaled CO (an oxidative breakdown product of heme and thus a marker of oxidative stress) is easily measured but may not be a useful biomarker as it is only slightly elevated in COPD and its measurement is confounded by active and passive smoking (9). Exhaled ethane levels are elevated in COPD and correlate with disease severity (10); however, the current assay methodology is too complex for routine assessment.

Exhaled breath condensate is another noninvasive and simple sampling technique for inflammatory mediators in the lung, including oxidative products, leukotrienes, cytokines, and pH (which reflects tissue acidification due to inflammation) (6). Limitations associated with exhaled breath condensate biomarkers include wide variability, due largely to dilution from water vapor during condensation, and low concentrations of the biomarkers that approach the detection limits of the assays. These limitations require correction and validation before exhaled breath condensate can be reliably used to assess disease progression in patients with COPD.

Biomarkers in plasma and serum (interleukin [IL]-6, IL-8, tumor necrosis factor [TNF]-α, fibrinogen, and Creactive protein [CRP]) have been studied for their relationship to disease severity in COPD (11). A recent meta-analysis demonstrated that neither serum CRP nor serum TNF-α levels are statistically significantly different between healthy subjects and patients at different COPD stages (11). Data on the relationships between serum IL-6, IL-8, or fibrinogen and COPD are insufficient and inconclusive.

In summary, biomarkers obtained from noninvasive collection methods offer future promise for assessing disease progression. However, methodological improvements and validation of the relationship between the biomarkers and disease severity are required before these markers can be widely adopted as surrogates of COPD disease progression.

### Physiologic indices as surrogate markers for disease progression in COPD

**Figure 3:**
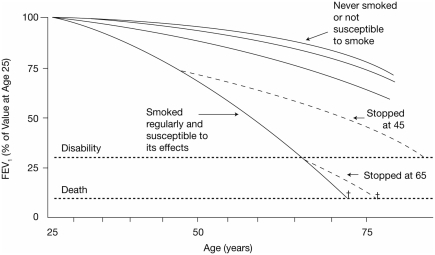
Natural history of lung function decline in smokers and nonsmokers (13). British Medical Journal, 1977,1,1645–1648, reproduced/amended with the permission from the BMJ publishing group.

Subtle physiologic changes that may reflect early pathology involving peripheral airways occur relatively early in the course of cigarette smoking (12), but may or may not progress to clinically significant physiologic impairment. Abnormalities in airflow (measured by FEV_1_/FVC) are considered the physiologic hallmark of COPD by which the disease is defined. It is well known that FEV_1_ declines with age in all adults aged 25 years and older; however, the rate of decline is more rapid in the susceptible smoker than in age-matched healthy nonsmokers (Figure 3) (13). Just as changes in FEV_1_ lag behind inflammatory and tissue changes, the development of symptoms is also delayed in comparison with significant decrements in FEV_1_. In fact, dyspnea may not become evident until FEV_1_ has declined to 50–60% of predicted normal (14). Thereafter, symptoms continue to increase at a rate that inversely correlates with the subsequent annual change in FEV_1_ (14).

**Figure 4:**
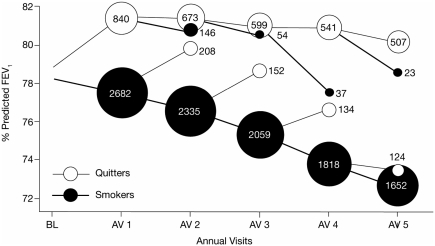
Effect of smoking cessation on the decline in lung function (forced respiratory volume in 1 second [FEV_1_] % predicted) over 5 years (16). Scanlon PD, Connett JE, Waller LA, Altose MD, Bailey WC, Buist AS; Lung Health Study investigators/ 2000/ Smoking cessation and lung function in mild-to-moderate chronic obstructive pulmonary disease/ American Journal of Respiratory and Critical Care Medicine/ 161/381–390. Official Journal of the American Thoracic Society © American Thoracic Society.

The rate of FEV_1_ decline is not easily modified by interventions. It is therefore unclear whether this index is resistant to change or if the approaches to its modification that have been assessed so far are ineffective. To date, only smoking cessation unequivocally decreases the rate of FEV_1_ decline (Figure 4) (13, 15, 16). Pharmacologic interventions, such as inhaled corticosteroids (ICS), which are the “gold standard” anti-inflammatory agents for controlling inflammation in asthma, have failed to alter the rate of FEV_1_ decline reported in large trials lasting 3 years or more (15, 17–19). Meta-analyses of these trials either confirmed the original negative results (20, 21) or identified a modest but statistically significant 7.7 mL per year improvement in the annual rate of FEV_1_ decline – the clinical significance of which is uncertain (22). Similarly, the antioxidant and mucolytic *N*-acetylcysteine failed to demonstrate any impact on the rate of FEV_1_ decline over 3 years, despite the anticipated value of this agent given that oxidative stress is believed to play an important role in COPD pathogenesis (23).

**Figure 5:**
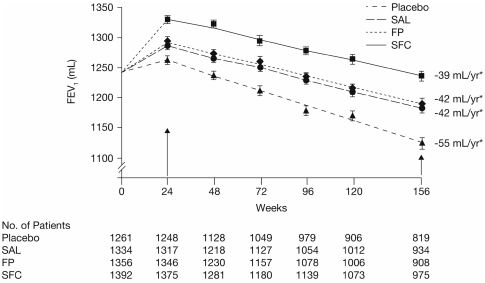
Effect on forced respiratory volume in 1 second (FEV_1_) of salmeterol and fluticasone propionate administered either alone or in combination versus placebo over 156 weeks (24). Calverley PM, Anderson JA, Celli B, Ferguson GT, Jenkins C, Jones PW, Yates JC, Vestbo J; TORCH investigators. N Engl J Med 2007;356:775–789. Copyright © [2007] *Massachusetts Medical Society. All rights reserved*.

**Figure 6:**
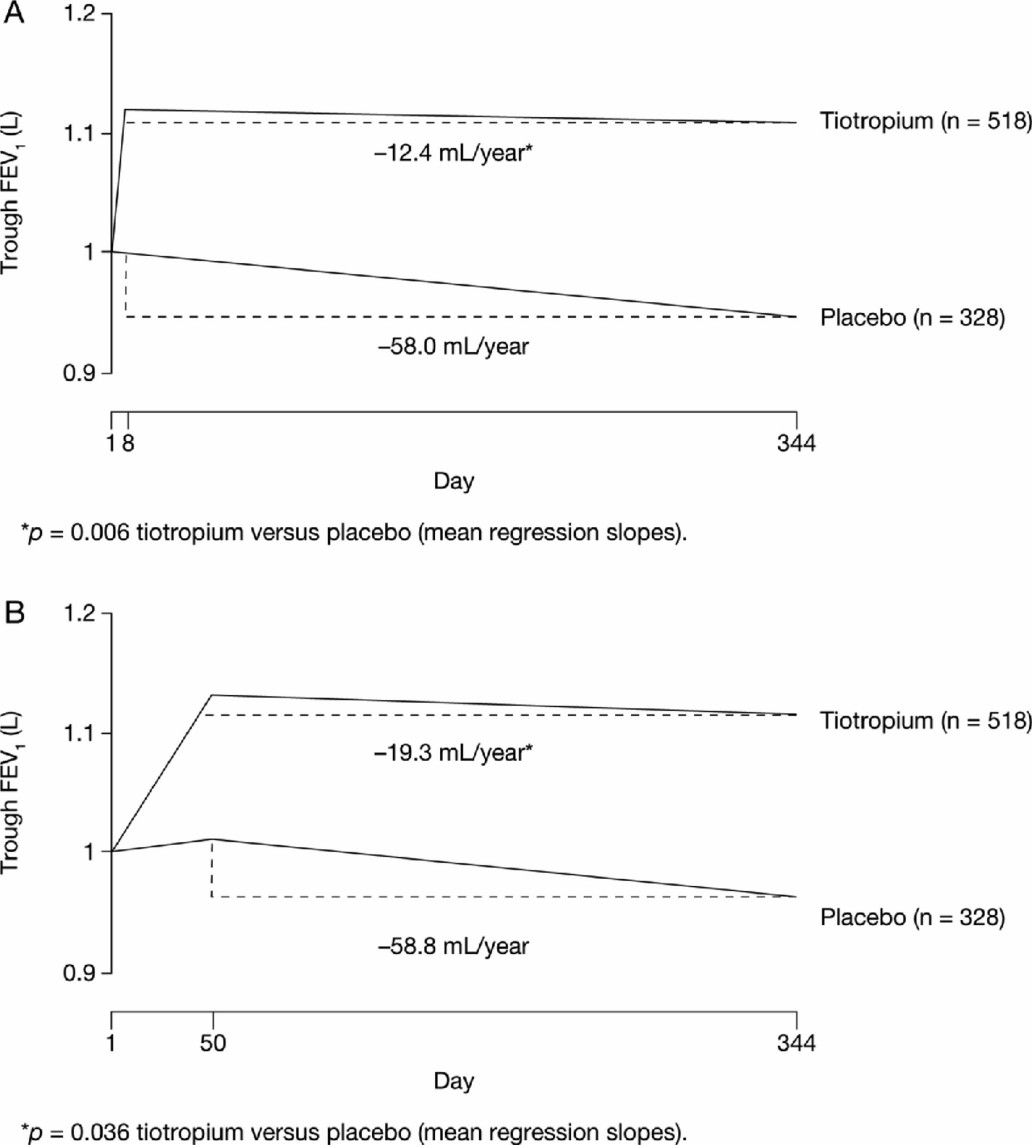
Effect of tiotropium on trough forced expiratory volume in 1 second (FEV_1_) following treatment with tiotropium or administration with placebo from (A) Days 8–355 and (B) Days 50–344 (26). Reprinted from Pulmonary Pharmacology and Therapeutics, 18, Anzueto A, Tashkin D, Menjoge S, Kesten S, One-year analysis of longitudinal changes in spirometry in patients with COPD receiving tiotropium, 75–81., Copyright (2005), with permission from Elsevier.

Only a few trials appear to have shown that pharmacological intervention alters the rate of FEV_1_ decline and, thereby highlight the usefulness of this index in characterizing disease progression. The TOwards a Revolution in COPD Health (TORCH) trial evaluated the ability of pharmacologic interventions (salmeterol/fluticasone combination, fluticasone alone, salmeterol alone, or placebo) to alter all-cause mortality in approximately 6,000 patients with moderate to very severe COPD over the course of 3 years (24). The decline in the rate of FEV_1_ was not a primary or secondary endpoint and centralized spirometry was not employed; however, post-hoc analysis suggests that the rate of decrease in postbronchodilator FEV_1_ between 6 months and 3 years was slightly but significantly less with salmeterol/fluticasone (39 mL per year; *p* < 0.001) and with either fluticasone alone or salmeterol alone (both 42 mL per year; *p* = 0.003), compared with placebo (55 mL per year) (Figure 5), with no differences among active treatment arms (25). Similarly, post-hoc evaluation of FEV_1_ data from a 1-year placebo-controlled study of tiotropium in COPD revealed a rate of decline in predose FEV_1_ with tiotropium of 12.4 mL per year (n = 518) compared with a 58 mL per year decline with placebo (n = 328) (*p* = 0.005) (Figure 6) (26). A lower and nonsignificant difference in the slope of decline in postbronchodilator FEV_1_ was also noted.

Based on these data, a large-scale, multinational trial (Understanding Potential Long-term Impacts on Function with Tiotropium [UPLIFT]) of nearly 6,000 patients with moderate-to-severe COPD (FEV_1_ <70% of predicted normal after maximal bronchodilation) was conducted to determine whether the long-acting antimuscarinic tiotropium unequivocally decreases the rate of both pre- and postbronchodilator FEV_1_ decline in COPD over a 4-year period in the context of freely prescribed medications for the treatment of COPD except inhaled anticholinergics (27). The study also examined whether treatment-related reduction in the rate of FEV_1_ decline is associated with concomitant improvements in patient-centered outcomes (health status, exacerbations, hospitalizations for COPD), as well as all-cause mortality. The findings indicated that although tiotropium was associated with sustained improvements in lung function, quality of life, and exacerbations during a 4-year period, it did not significantly reduce the rate of decline in FEV_1_ (27). These negative findings need to be interpreted in the light of the fact that over 70% of UPLIFT participants used an inhaled corticosteroid and/or long-acting beta-agonist during the course of the trial (27), thus making it more difficult to demonstrate an impact of tiotropium on top of any potential effect of these concomitant therapeutic agents on decline in FEV_1_.

One limitation that affects all of the long-term studies, including TORCH and UPLIFT, is that many patients fail to complete the studies, with significantly more patients withdrawing from the placebo arms than the active treatment arms (25, 27). It has been observed that the patients who withdraw are often those who are experiencing more rapid deterioration in lung function, thereby resulting in the remaining patients essentially representing “healthy survivors” (25, 27). The higher drop-out rate for individuals in the placebo arm with poorer lung function potentially minimizes the differences in lung function decline observed between placebo and active interventions (25, 27).

### Patient-centered outcomes as surrogate markers for disease progression in COPD

#### Dyspnea

Dyspnea may be considered a potential marker of disease progression in COPD because of its link with lung function, the manner in which it changes over time, its utility in predicting mortality, and its responsiveness to therapy. Dyspnea resulting from dynamic and resting hyperinflation in COPD is now increasingly recognized as a clinically important determinant of exercise limitation and the ability to perform activities of daily living (28).

Findings from a range of studies suggest that dyspnea worsens gradually over time in stable COPD. For example, in a study in which changes in dyspnea were systematically assessed using the UCSD Shortness of Breath Questionnaire in patients undergoing pulmonary rehabilitation or education, dyspnea improved or remained stable in most patients, irrespective of the intervention over the first 4 years of the study; however, dyspnea worsened in both groups over the following 2 years (29).

In a 1-year placebo-controlled trial with tiotropium in patients with moderate to very severe COPD, dyspnea (measured with the Transition Dyspnea Index [TDI]) in the placebo group improved over the first 50 days then worsened (30). In another study in patients with stable but symptomatic COPD, dyspnea (measured by TDI) worsened over time and, interestingly, inspiratory muscle strength also declined (*p* < 0.001). Multiple factors, including but not limited to worsening pulmonary mechanics resulting from respiratory muscle weakness, may be responsible for these increases in dyspnea. In addition, increased dyspnea is reflected by deterioration in physical functioning, which may lead patients to reduce physical activity in order to minimize discomfort, thereby further impairing exercise tolerance and quality of life.

Many long-term trials of pharmacotherapy in COPD have assessed the impact of interventions on dyspnea. Salmeterol/fluticasone fixed combination therapy, when compared with placebo, was found to produce sustained improvements in TDI score over 6 months that were statistically and clinically significant (+1.7 unit difference) (31) or statistically significant but less than the minimal clinically important difference (+0.8 difference) (32). Similarly, using a Likert scale to grade the degree of breathlessness, treatment with long-acting β-agonist (LABA)/ICS combinations has resulted in statistically significant reductions in dyspnea (*p* < 0.001) relative to placebo in 1-year trials (33–35). The long-acting anticholinergic tiotropium produced improvements in dyspnea (measured by the TDI) that were both statistically and clinically significant compared with placebo (+1.14 difference at 1 year) (30). These improvements were sustained with tiotropium whereas dyspnea worsened progressively in the placebo arm over the year.

In conclusion, dyspnea worsens over time in COPD, is related to physical activity and quality of life, and exhibits sustained improvement with pharmacologic and nonpharmacologic interventions; therefore, dyspnea could be considered a surrogate marker for disease progression in COPD.

#### Exercise capacity

Exercise intolerance and exertional dyspnea are major symptoms of COPD and worsen as the disease progresses. The pathophysiologic basis for the accelerated decline in exercise performance is rooted in airflow obstruction that results in air trapping and hyperinflation, particularly under the dynamic conditions of exercise when an increased ventilatory requirement leads to tachypnea, which shortens the time available for exhalation. Static and dynamic hyperinflation, in turn, lead to exertional dyspnea and impaired exercise performance due to the associated reduction in inspiratory capacity and restricted ability to expand the tidal volume to meet the increased ventilatory demands of exercise.

**Figure 7:**
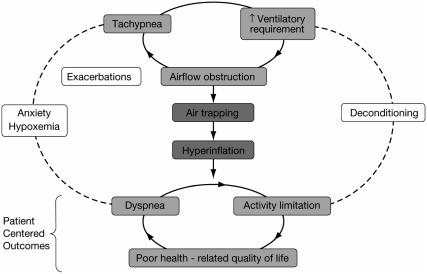
Air trapping links pathophysiology and patient-centered outcomes in COPD (37). Reprinted from American Journal of Medicine, 119, Cooper CB, The connection between chronic obstructive pulmonary disease symptoms and hyperinflation and its impact on exercise and function, S21–S31., Copyright (2006), with permission from Elsevier.

Gas-exchange abnormalities, including hypoxemia and increased wasted ventilation fraction, also contribute to exercise limitation. An important consequence of the exercise impairment in COPD is adoption of a more sedentary lifestyle and consequent physical deconditioning that results in lactic acid accumulation from premature anaerobiosis during exercise. The latter, in turn, stimulates ventilation and further reduces the time for exhalation, thereby leading to a vicious cycle of additional air trapping, dyspnea, and exercise limitation (36). Ultimately, this cycle leads to a decline in health-related quality of life (HRQoL) (Figure 7) (37).

Although it is well known that severe COPD limits exercise capacity, even mild airflow limitation has been shown to reduce maximal exercise performance. One study demonstrated that patients with mild airflow obstruction (FEV_1_ 74–78% of predicted) due to COPD had significant reductions in peak oxygen uptake (VO_2max_) (38). Interestingly, these reductions in VO_2max_ were related to reductions in tidal volume during maximal exercise that most likely resulted from a reduced inspiratory capacity due to dynamic hyperinflation, although the latter factors were not directly measured. Another study of patients with mild-to-moderate COPD (mean FEV_1_ 72% of predicted normal) demonstrated significantly reduced VO_2max_ during exercise (69% of predicted) that was associated with the development of dynamic hyperinflation during exercise, despite the presence of a normal end-expired lung volume at rest (39). These findings demonstrate the importance of dynamic hyperinflation in exercise impairment for all COPD severities.

Exercise tolerance shows variable improvement with different interventions in COPD, depending both on the nature of the intervention and on the test used to measure exercise performance. The 6-minute walking distance (6MWD) can be confounded by poor repeatability and learning effects, but exercise endurance time during a constant work rate submaximal exercise test has been shown to be a reliable and repeatable measure of exercise endurance that is responsive to changes with bronchodilator therapy in COPD (40, 41). Pulmonary rehabilitation generally improves exercise endurance but has little effect on maximal exercise performance (42). Bronchodilator therapy has only infrequently been shown to improve the 6MWD but has more often been observed to improve submaximal exercise endurance time (43–45).

Oga and colleagues (46) compared the sensitivity of 3 different exercise tests—6MWD, incremental cycloergometry (ICE), and endurance time—to detect changes with anticholinergic bronchodilator therapy. While all three tests revealed a statistically significant improvement in exercise performance after administration of the bronchodilator (compared with placebo), the percent improvement was substantially greater when exercise performance was measured by endurance time (19%) than by VO_2max_ (3%), maximal minute ventilation (6%), maximal work rate (4%), or 6MWD (1%). Therefore, the endurance test appears to be the most sensitive method for assessing the benefits of an intervention on exercise performance in COPD.

Exercise capacity also appears to be sensitive to therapeutic intervention. Tiotropium administered daily for 6 weeks progressively improved endurance time compared with placebo during a constant work rate exercise test (43, 44). These beneficial effects on exercise performance were paralleled by improvements in inspiratory capacity, tidal volume, minute ventilation, and dyspnea. Salmeterol also led to improvements in several measures of exercise performance during both incremental and constant-load exercise after 2 weeks of treatment (45). The combination of pulmonary rehabilitation and treatment with tiotropium has been shown to have additive effects in improving exercise tolerance in COPD.

These improvements are presumably related to improved muscle conditioning resulting from the pulmonary rehabilitation and reduced airflow limitation and hyperinflation concomitant with bronchodilator treatment (47). The benefits of combined pulmonary rehabilitation and tiotropium in improving exercise tolerance were accompanied by clinically meaningful improvements in dyspnea and quality of life compared with pulmonary rehabilitation alone. Strategies that lead to substantial improvements in exercise tolerance or reduce its rate of decline, such as long-acting bronchodilator treatment in combination with pulmonary rehabilitation, could therefore be considered to be disease-modifying.

#### Health-related quality of life

Placebo arms of randomized controlled trials as well as cohort studies demonstrate that HRQoL declines with time in patients with COPD (48, 49). Numerous studies have demonstrated correlations between decline in HRQoL and other aspects of COPD, such as exacerbations, FEV_1_ decline, and mortality. For example, data from the ISOLDE (Inhaled Steroids in Obstructive Lung Disease) trial demonstrated correlations between exacerbation rate and HRQoL (50). Frequent exacerbations (>1.65 per year) were independently associated with a worse baseline St George's Respiratory Questionnaire (SGRQ) score and a more rapid rate of deterioration in HRQoL. Similarly, in the same trial, progressive change in FEV_1_ was observed to correlate with change in SGRQ score (49). Poor HRQoL has also been shown to predict mortality (51).

**Figure 8:**
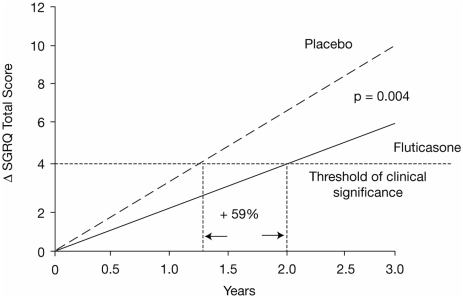
Slope of deterioration in health status calculated using estimates from a random coefficients hierarchical model for patients treated with fluticasone proprionate or placebo over 3 years (49). Spencer S, Calverley PM, Sherwood Burge P, Jones PW; ISOLDE Study Group/ 2001/ Health status deterioration in patients with chronic obstructive pulmonary disease/ American Journal of Respiratory and Critical Care Medicine/ 163/ 122–128. Official Journal of the American Thoracic Society © American Thoracic Society.

**Figure 9:**
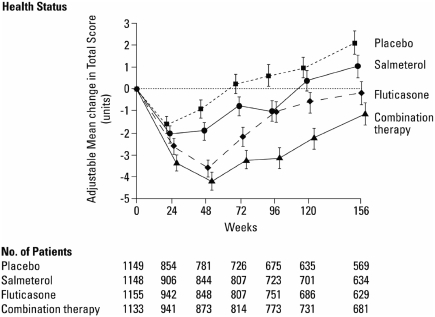
Change in Health-related Quality of Life (HRQoL) over 156 weeks following treatment with placebo, fluticasone proprionate, salmeterol or fluticasone proprionate and salmeterol administered in combination (24). Calverley PM, Anderson JA, Celli B, Ferguson GT, Jenkins C, Jones PW, Yates JC, Vestbo J; TORCH investigators. N Engl J Med 2007;356:775–789. Copyright © [2007] *Massachusetts Medical Society. All rights reserved*.

The rate of change in HRQoL can be altered by therapeutic interventions. For example, SGRQ total scores in patients with moderate to severe COPD treated with fluticasone propionate 500 μg twice daily took longer to deteriorate by 4 points (the minimum clinically important difference) than patients treated with placebo (24 months versus 15 months) (Figure 8) (49). Similarly, over a period of 1 year, patients with stable COPD who were treated with tiotropium 18 μg showed sustained improvements in their HRQoL scores whilst patients receiving placebo showed the expected decline in health status (30). Combination treatment with salmeterol and fluticasone also produced a clinically significant improvement in HRQoL and lung function compared with placebo in patients with COPD (Figure 9) (24).

These results must be considered while bearing in mind the effects of COPD exacerbations on HRQoL. It is well known that HRQoL deteriorates significantly following an exacerbation (52). Thus, the extent of improvement in HRQoL seen for interventions that reduce the rate of exacerbations will differ from that of interventions that only improve other aspects of the disease such as dyspnea or FEV_1_. In addition, results from placebo-controlled trials are likely to be influenced by differential drop-out rates between groups receiving active treatment and those on placebo. Analysis of HRQoL in the ISOLDE study revealed that the deterioration in SGRQ total, symptom, and impact scores for placebo-treated patients who withdrew was significantly greater than either that of placebo completers or the patients treated with fluticasone (53).

When considering whether changes in HRQoL are an appropriate means by which to monitor disease progression, it is important to recognize that HRQoL measures reflect the effects of the disease, rather than providing a measure of the disease itself. The relationship between poor HRQoL and other disease aspects, e.g., increased mortality, results from the fact that both reflect underlying disease activity. One of the advantages of considering changes in HRQoL as a surrogate marker for disease progression is that it can be monitored relatively easily using disease-specific questionnaires.

#### Exacerbations

Exacerbations are primarily a feature of severe-to-very-severe COPD, although they also appear in the earlier stages of the disease. Frequent exacerbations are associated with more rapid decline in FEV_1_, impaired functional status and HRQoL, worse survival, and increased costs and hospitalizations (50, 54–56).

A significant body of evidence supports the use of LABAs, long-acting muscarinic antagonists, and ICS to reduce the rate of exacerbations in patients with moderate-to-severe COPD (34, 35, 57). Furthermore, combining these agents may provide greater benefits than use of each component alone. For example, recent data from the TORCH study suggest that the combination of a LABA and an ICS reduces the rate of exacerbations to a significantly greater extent than either component administered alone (*p* < 0.002 for salmeterol and fluticasone versus salmeterol; *p* < 0.02 for salmeterol and fluticasone versus fluticasone) (24). Reducing or preventing exacerbations is an important component of disease modification in COPD; however, the low frequency of exacerbations in patients with mild COPD may limit the utility of this outcome as a measure of progression during the early stages of the disease.

### Mortality

Death is the ultimate consequence of disease progression and thus reduced mortality and improved survival describe an impact on disease progression. Numerous factors are reported to influence mortality in COPD including FEV_1_ (58), dyspnea (59), disease duration (60), carbon dioxide and oxygen arterial tensions (56), cardiac status (54), body mass index (61), serum albumin level (62), functional status (63), exercise limitation (63, 64) and comorbidities (55). The correlations between these cofactors and long-term mortality are variable.

**Figure 10:**
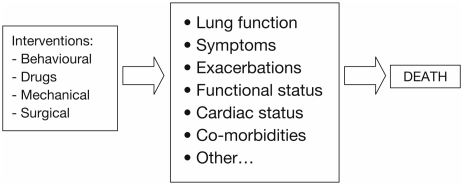
Direct and indirect ways by which interventions impact on mortality.

Interventions that impact on mortality most probably do so via improvements in aspects of lung function and patient-centered outcomes, although there may be other, as yet unknown ways in which interventions affect this endpoint (Figure 10). Smoking cessation has beneficial effects on subsequent mortality, as demonstrated by an 18% reduction in all-cause mortality after 14.5 years (65). Long-term home oxygen therapy has been shown to reduce mortality in patients with persistent hypoxemia, as has lung volume reduction therapy in highly selected patients with emphysema (66–68). In addition, a recent meta-analysis showed that pulmonary rehabilitation after a COPD exacerbation reduced the risk of mortality (pooled relative risk 0.45 [95% confidence interval (CI) 0.22–0.91]) (69). Further research is required to define the long-term effects of these interventions.

The effect of a pharmacologic intervention on mortality was recently investigated in the TORCH study. In this study, patients received either salmeterol/fluticasone in combination, salmeterol alone, fluticasone alone, or placebo, for 3 years (24). Of 6,112 patients in the efficacy population, 875 died over the 3-year study. The hazard ratio for all-cause mortality in the combination therapy group compared with the placebo group was 0.825 (95% CI 0.681–1.002; *p* = 0.052), corresponding to a reduction in the absolute risk of death of 2.6% (17.5% relative reduction), which approached statistical significance. Further factorial analysis indicated that the effect on mortality of the combination therapy appeared to be entirely due to salmeterol, and that the effect was highly significant (*p* = 0.004) (70).

This underscores the important role that airflow limitation plays in disease progression and, consequently, the need for it to be managed. Despite the lack of a statistically significant reduction in mortality, combination therapy did result in significantly fewer exacerbations and improved HRQoL and lung function compared with placebo. Clinical findings from the study, however, suggest that monotherapy with corticosteroids should not be recommended, but that LABAs used alone or in combination may provide benefit. It is clear from the findings of the TORCH study that further investigation is required into the effect on mortality of pharmacotherapy in COPD.

While UPLIFT focused primarily on the rate of decline in FEV_1_ as a primary endpoint, mortality was a key prespecified secondary endpoint. As in TORCH, vital status was ascertained in nearly all patients over the protocol-defined treatment period, permitting an intention-to-treat analysis of the impact of the study medication on mortality. The hazard ratio for all-cause mortality over the 1,440-day protocol-defined duration of the study (vital status known in 95% of all randomized subjects) was 0.87 (95% CI 0.76–0.99; *p* = 0.034), while that over this same time period plus 30 days (1470 days), as prespecified in the analysis plan, (vital status known in only 75% of all subjects over the latter time period) was 0.89 (95% CI 0.79–1.02; *p* = 0.086) (27).

Thus, both of the recent long-term trials of pharmacotherapy in COPD (TORCH and UPLIFT) revealed an impact on mortality that came tantalizingly close to achieving statistical significance. The mechanism(s) of this effect need to be further explored but could involve several factors, including reductions in exacerbations and in respiratory failure, as well as improvements in ventilatory mechanics, including a reduction in hyperinflation, that may have indirect cardiac benefits through, for example, a decrease in cardiac afterload.

Since mortality is an end result of COPD, it can be a useful endpoint to define an impact on disease progression within a population receiving a study medication. However, mortality is not suitable for assessment of disease progression in individual patients. It is further complicated by comorbid conditions associated with COPD that can involve organ system dysfunction sufficient to contribute to mortality independently or additionally, but not exclusively due to COPD. Therefore, mortality is appropriate to define the end result of disease progression; however, it is not appropriate for describing disease progression in individual patients or in clinical practice.

## CONCLUSIONS

The proposed working definition of disease modification was:

an improvement in, or stabilization of, structural or functional parameters as a result of reduction in the rate of progression of these parameters which occurs whilst an intervention is applied and may persist even if the intervention is withdrawn.

In many cases the structural changes cannot be monitored directly and surrogate markers of improvements must be used. These remain to be fully delineated, but include physiological parameters, such as FEV_1_, as well as patient-centered outcomes, such as exacerbation rates, breathlessness, exercise tolerance, and HRQoL. An important unanswered question concerns the minimum duration of the structural/functional improvement that is required for an intervention to be considered disease-modifying. Longitudinal data are needed to refine the definition of disease modification in COPD.

FEV_1_ decline retains an important role in monitoring the course of COPD. However, given the interdependence between physiological and patient-centered outcomes in COPD, treatments aimed at preventing disease progression should ideally demonstrate improvements across multiple outcomes. According to the proposed definition, smoking cessation and lung volume reduction surgery should be considered to be disease-modifying interventions. A pharmacologic intervention may be considered disease-modifying if it provides consistent and sustained improvements in structural (e.g., reduction in exacerbations as surrogate marker) and functional (e.g., improvement in FEV_1_ and exercise tolerance as surrogate marker) parameters.
